# Team Reasoning and Collective Intentionality

**DOI:** 10.1007/s13164-016-0318-z

**Published:** 2016-08-05

**Authors:** Björn Petersson

**Affiliations:** Department of Philosophy, Lund University, Lund, Sweden

## Abstract

Different versions of the idea that individualism about agency is the root of standard game theoretical puzzles have been defended by Regan 1980, Bacharach (*Research in Economics* 53: 117–147, 1999), Hurley (*Behavioral and Brain Sciences* 26: 264–265, 2003), Sugden (*Philosophical Explorations* 6(3):165–181, 2003), and Tuomela 2013, among others. While collectivistic game theorists like Michael Bacharach provide formal frameworks designed to avert some of the standard dilemmas, philosophers of collective action like Raimo Tuomela aim at substantive accounts of collective action that may explain how agents overcoming such social dilemmas would be motivated. This paper focuses on the conditions on collective action and intention that need to be fulfilled for Bacharach’s “team reasoning” to occur. Two influential approaches to collective action are related to the idea of team reasoning: Michael Bratman’s theory of shared intention and Raimo Tuomela’s theory of a we-mode of intending. I argue that neither captures the “agency transformation” that team reasoning requires. That might be an acceptable conclusion for Bratman but more problematic for Tuomela, who claims that Bacharach’s results support his theory. I sketch an alternative framework in which the perspectival element that is required for team reasoning - the ‘we-perspective’ - can be understood and functionally characterized in relation to the traditional distinction between mode and content of intentional states. I claim that the latter understanding of a collective perspective provides the right kind of philosophical background for team reasoning, and I discuss some implications in relation to Tuomela’s assumption that switching between individual and collective perspectives can be a matter of rational choice.

## Introduction

Different versions of the idea that individualism about agency is the root of standard game theoretical puzzles have been defended by Regan 1980, Bacharach 1999, Hurley 2003, Sugden 2003, and Tuomela 2013, among others. While collectivistic game theorists like Michael Bacharach provide formal frameworks designed to avert some of the standard dilemmas, philosophers of collective action like Raimo Tuomela aim at substantive accounts of collective action that may explain how agents in such frameworks would be motivated.

The idea that acknowledging genuinely collective agency is the key to eliminate well-known game theoretical problems like the prisoners’ dilemma is sometimes presented as a challenge to standard game theory.

The source of these problems of coordination and cooperation is not the nature of the individuals’ goals, or the instrumental character of rationality. Rather it is individualism about rationality, which holds the unit of activity exogenously fixed at the individual. (Hurley 2004, 201)

However, it is fair to stress from the start that if we regard the two players in a prisoners’ dilemma as a unified team or a single agent, we have chosen to look at another game. If [I & you] is a unified agent choosing between the four alternatives in a matrix that would have illustrated a prisoner’s dilemma for you and me as two individual agents, this collective agent is not facing a prisoners’ dilemma. Moving from individual agents to collective agents will not affect the game theorist’s verdict. So, the collective move does not really challenge game theory in that respect, and there is no need for doing so.

Game theorists will never accept the idea that collective rationality might ensure cooperation in one-shot prisoners’ dilemmas (Binmore 2007, 265). On the other hand, game theorists willingly admit that people have the capacity to change the game in a variety of ways. Two real-life prisoners may do so when time for interrogation approaches by explicitly binding themselves to a deal, thereby subjecting themselves “to the penalty of never being trusted again in the case of failure” (Hume 1739, 3:2.5) or worse, depending on the future sanctions for deal-breaking that are available to them. That may change their preferences and thereby avert the prisoners’ dilemma situation by altering the payoffs. They might also change the game by starting to think about the likelihood that they will need to face a similar situation together again, perhaps more than once. Then they may conceive of the situation as an indefinitely iterated prisoners’ dilemma that might have other rationally admissible strategies.^1^ Etc.

In a similar way, the move to a collective perspective would be a way of transforming the choice situation, not an attempt at solving the prisoners’ dilemma. We might say that were it not for their capacity for collective decision-making, the two prisoners would have been facing a prisoners’ dilemma. However, there is a significant difference between this type of transformation of the potential prisoner’s dilemma situation, and the sort of change that occurs when promises and sanctions (or moral considerations, altruistic preferences, or other substantial assumptions about the players’ psychology) are introduced. Sanctions affect the situation by altering the individual payoffs, i.e. changing the individual preferences. The game-theoretical notion of *team reasoning* need not in itself involve any psychological assumptions about how people are motivated. Game theory in general is neutral about what people want and just describes what they should do to maximize their payoffs, i.e. whatever it is that they want.

Central to the theory of team reasoning is the idea that players may *frame* a choice situation with given individual payoffs in two different ways: I may frame a situation from my individual viewpoint, or from my team’s viewpoint. The possibility of *vacillation* between these frames is essential to capture what may happen when players are confronted with a potential social dilemma. How realistic is this assumption?

The relation between substantive philosophical theories of collective intentionality and the game theoretical notion of team reasoning can perhaps be regarded as parallel to the relation between (Humean, belief-desire model) philosophy of action and the decision theoretical conception of individual reasoning. In both cases, the formal model can in principle do without substantial assumptions about the essential features of human motivation. Nevertheless, substantive philosophical theories about the nature of (individual or collective) motivation provide a sort of underpinning that may give the formal theory greater explanatory and normative force. Alternatively, substantive theories may convince us that the formal model’s conditions on agency and decision-making require too much idealisation or simplification for the model to give any guidance or be of any greater explanatory value when it comes to real life decision-making.^2^

The issue I want to examine here is the conditions on collective action and intention that need to be fulfilled for team reasoning of the right kind to occur. Section 2 presents informally the main features of Michael Bacharach’s team reasoning in relation to some standard game theoretical puzzles. Sections 3 and 4 relate two distinct influential approaches to collective intentions and actions to the idea of team reasoning: Michael Bratman’s theory of shared intention and Raimo Tuomela’s theory of a we-mode of intending.^3^ I agree with Tuomela that Bratman’s theory will not make room for team reasoning in Bacharach’s sense, but I am not sure that such a limitation has to be problematic for Bratman. Tuomela explicitly claims that his theory accounts for the agency transformation that Bacharach’s team reasoning requires, and hence that Bacharach’s results support his theory. I show that Tuomela’s explicit analyses of ‘we-mode’ fail to capture the perspectival element that is essential in his applications of the concept. Section 5 sketches an alternative framework in which this perspectival element – the ‘we-perspective’ - can be understood in relation to the traditional distinction between mode and content of intentional states.^4^ In section 6 I claim that the latter understanding of a collective perspective provides the right kind of philosophical background for team reasoning, and I discuss some implications in relation to Tuomela’s assumption that switching between individual and collective perspectives can be a matter of rational choice.

## Team Benefactors and Team Reasoners

Michael Bacharach distinguishes between two ways in which collectivity may enter individual motivation when players are confronted with potentially problematic social choices. Roughly, a *team benefactor* is an individual player who is motivated by concern for her own team’s well-being, while a *team reasoner* in addition frames the decision problem from her team’s perspective.

**Fig. 1:**
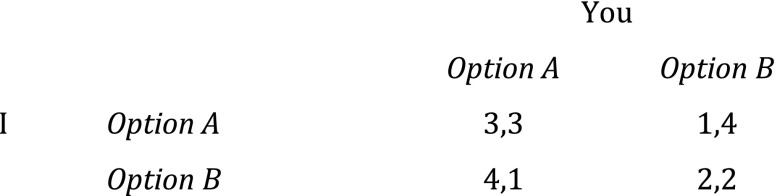
The prisoner’s dilemma (1)

Suppose two self-caring individuals, you and I, are faced with a standard prisoner’s dilemma of the following form (numbers represent size of payoffs as determined by our individual preferences, and “4,1” means that I, the row player, get 4 and that you, the column player (Fig. 1), get 1):^5^

**Fig. 2:**
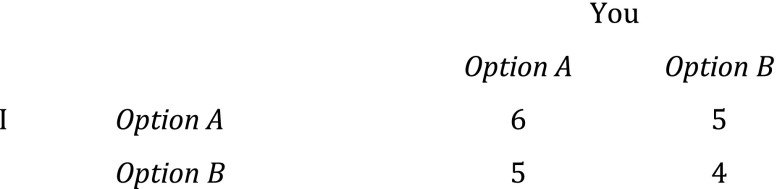
The team benefactor’s delight. (”Team Benefactor’s Delight” alludes to Binmore’s ”Prisoner’s Delight”, where players prefer option *A* because they love each other. (Binmore 2007, 12))

Rationality dictates for each self-caring individual that she should pick *B* regardless of what the other one does.^6^ Now, assume for simplicity that you regard the *group’s* well-being as equal to the sums of individual payoffs from the original prisoners’ dilemma – i.e. that your preferences for our group are in effect preference-utilitarian – and that you are a team benefactor, a person who wants the best for our group.^7^ The resulting matrix will look like this to you (Fig. 2).

Then you will choose *A* whatever I do. If you regard me as a similar kind of team benefactor, you will also expect me to choose *A* before *B*. Given how the utilities are distributed, that expectation is not a condition for the rationality of your choice though. The utility for *us* in terms of preference satisfaction will be higher if you pick *A* even if I take *B*. So, sometimes groups of team benefactors will achieve collectively attractive outcomes in situations that would have been prisoners’ dilemmas for ordinary self-caring agents.

**Fig. 3:**
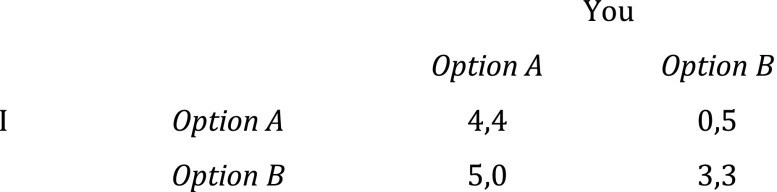
Prisoners dilemma (2)

However, as Bacharach shows, adopting team benefactor reasoning will not always guarantee the collectively attractive outcome. This kind of preference transformation will not even guarantee that result in all potential prisoners’ dilemmas. Suppose our initial individual self-regarding preference orderings were slightly different, as illustrated (Fig. 3).

**Fig. 4:**
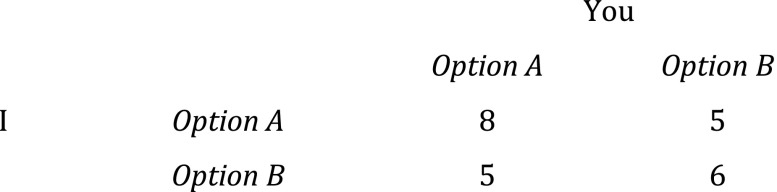
Team benefactor’s Hi-Lo

This is still a genuine prisoners’ dilemma, where it is rational for you to choose *B* regardless of what I do, and vice versa. If each player then becomes a team benefactor of the kind that wants to maximize the satisfaction of the group members’ initial self-regarding preferences the game will change, but in this case not in a way that guarantees the outcome that is best in terms of satisfaction of the group members’ initial preferences. For such a team benefactor, the matrix would look like this (Fig. 4).

As team benefactors we would be facing a Hi-Lo game instead of the prisoners’ dilemma we were in before we started caring about the group. The unfortunate thing about being in a Hi-Lo game is that rationality will not help us achieve what appears to be the collectively best alternative. Rationality dictates nothing more than that I should choose *A* if you choose *A* and *B* given that you pick *B*, and vice versa for you. If I choose *A* when you choose *B*, I do things worse for us in utilitarian terms.

Bacharach’s *team reasoner* approaches the situation not by asking what I should do, or what I should do for us, but what *we* should do. The latter question is not equivalent to any of the two first questions (as it might sometimes be in ordinary language) — it is a question about the selection of a profile, i.e. a set of strategies for the team members. From that selection of a profile the team reasoner then infers which action she should perform – her part in the collective action. The formal features of Bacharach’s team reasoning are designed to produce the collectively attractive result in the potential Hi-Lo game. Bacharach stresses a *we-perspective* and states that a form of *agency transformation* is required – the switch from being purely self-caring to becoming a team benefactor would be a mere preference transformation.

Bacharach acknowledges work done on we-thinking within the field of collective intentionality, and he refers to Raimo Tuomela and Margaret Gilbert. He makes clear that his aim is different though. He wants to provide a formal general model of team reasoning (Bacharach 1999, 119), while Tuomela and Gilbert provide substantive frameworks in which team reasoning may fit.

Theories of collective intentions can be treated as attempts to answer what Bacharach calls “the framing problem”. How would team reasoners have to conceive of their choice in order to transform the choice situation in the right way? Which conceptual resources would they have to employ? What are the phenomenological features of the team reasoner? What would be the proper expression of such attitudes?

Her frame stands to her thoughts as a set of axes does to a graph; it circumscribes the thoughts that are logically possible for her (not ever but at the time). In a decision problem, everything is up for framing /.../ also up for framing are her coplayers, and herself (2006, 69).

Bacharach assumes that team reasoners must at least have ‘we’ concepts in their frame. (Smerilli 2012, 544) However, ‘we’ and ‘have in their frame’ can be understood in different ways. Theories of collective intentionality give different answers on how to understand ‘we’ as it appears in the attitudes of the parties to collective actions. The notion can be understood reductively or non-reductively, for instance. Such theories also differ with respect to *how* the collective concept is supposed to belong in the attitude in question. Roughly, we can distinguish between theories like Michael Bratman’s, that place a concept of the group in the *content* of intentions, and theories like Searle’s or Tuomela’s, according to which collectivity in some sense is a feature of the manner in which the attitude is held.

## Bratman’s Shared Intentions

Raimo Tuomela suspects that if Bratman’s analysis captures the essence of collective intentionality, then we would at best be what Michael Bacharach calls “team benefactors” (Tuomela 2013, 159). I find that conclusion about Bratman’s view plausible. The core of Bratman’s theory is the *Intention condition* for collective intention.

*Intention condition*: We each have intentions that we *J*; and we each intend that we *J* by way of each of our intentions that we *J* (so there is interlocking and reflexivity) and by way of relevant mutual responsiveness in sub-plan and action, and so by way of sub-plans that mesh. (2014, 103)

The proper expression for the individual group member’s attitude is ‘I intend that we *J’,* where ‘we *J’* refers to a cooperatively neutral description of the joint activity which merely satisfies the “behavioural conditions” for cooperation. (That feature of the analysis is necessary to avoid vicious circularity.) Suppose we deliberate in these terms when we are about to decide upon our strategies in the potential dilemma situation. I assume that ‘to decide upon a strategy’ is equivalent to forming an intention to act in accordance with a strategy.

Unlike ‘we intend to *J’* phrases of the form ‘I intend that we *J’* are not commonly used in ordinary language, but if there is a question to which such a phrase is the answer, this seems to be a question I may ask myself when I am about to form my intention concerning us. Like in the intention that is expected to result from this deliberative process, ‘we’ figures in the *content* of the question but it is asked from my *perspective* rather than ours. In this framework, my intention that we do this rather than that would presumably result from what I want for us*,* my caring for how well we do. So, it seems reasonable to assume that the Bratmanian co-operator would not be asking what *we* should do in Bacharach’s distinct sense. Being a Bratmanian co-operator would not help in the potential Hi-Lo game.

Tuomela seems to assume that what makes Bratmanian co-operators behave like team benefactors rather than team reasoners is Bratman’s reductionist move to avoid circularity. (Tuomela 2013, 159). To achieve that Bratman makes clear that ‘we *J’* in the content of the co-operator’s intention cannot be required to refer to collective *J-ing* but merely to the purely behavioural counterpart to such an activity. Elsewhere I have expressed doubts about Bratman’s move for other reasons (Petersson 2007) but I do not think that understanding ‘we *J’* in terms of a substantial co-operatively loaded notion of collectivity in Bratman’s analysis will change much with respect to the Hi-Lo game and similar problems. Each player will still be acting on his preferences on behalf of the group, given beliefs about how others act upon their preferences on behalf of the group. The individuals might conceive of what is preferable for the group, or the group’s imagined action, in more substantially holistic collectivistic terms, but that need not change the game.^8^ Tuomela’s diagnosis of the Bratmanian co-operator’s failure at team reasoning blames her reductive conception of the group. I believe instead that the explanation of her failure is that the group merely figures in the content of her attitudes, and that this content is conceived from an individual perspective.^9^ No *agency transformation* occurs.

If Bratman’s approach is correct, the limits of collective intentionality are such that collective thinking will not help us in the Hi-Lo game. Bratman’s shared intentions will not suffice to avoid the deadlock.^10^ This does not necessarily discredit Bratman’s theory. Although Bratman’s complete theory of shared agency, which requires meshing sub-plans etc., may be designed to capture many kinds of social co-ordination, he has as far as I know not made any specific claims about shared intentions being the key to avoid Hi-Lo:s or other annoying games. The implication that they are not that key need not bother him. It might be an unfortunate fact that sometimes we have no direct rational clue to certain social dilemmas. Apparently fairly rational people fail to cooperate in real life on many occasions. And there are many other ways of changing the game, most prominently by introducing more or less explicit promises accompanied by sanctions. In games like Hi-Lo there may be evolutionary explanations of why certain reasonable strategies are conventionally favoured, etc. (Binmore 2007, 265). So, collective intentionality is just one among several possible explanations of why people sometimes get it right when confronted with potentially problematic social choices.

On the other hand, Bratman claims that

[c]onformity to social rationality norms that are central to shared intention—norms of social agglomeration, social consistency, social coherence, and social stability—will emerge from the norm-guided functioning of these interrelated attitudes of the individuals. Violation of such social norms will normally consist of a violation of associated norms of individual planning agency. (2014, 87)

As András Szigeti points out in a recent review, there appears to be a tension between these claims by Bratman and the lessons from game theory that indicate unavoidable clashes between individual and collective rationality. (Szigeti 2015) There are cases where being a co-operator in Bratman’s sense will not help avoiding such clashes, which the Hi-Lo game illustrates. Our ending up in the bottom right box, which seems socially irrational, does not violate any norms of individual planning agency.^11^

## Tuomela’s We-Mode

I claimed above that what makes Bratmanian co-operators unable to get it right merely in virtue of collective intentionality when faced with a potential Hi-Lo game is not the reductive understanding of the content of their co-operative intentions. The reason for their failure, instead, is that they necessarily conceive of the situation from the individual agent’s perspective. Roughly, the natural question to ask for such an agent would be “What should I do for us?” rather than “What should we do?” in the sense required for Bacharach’s team reasoning.

In his thorough discussion of Bacharach’s game theoretical results, Raimo Tuomela claims that “[Tuomelian w]e-mode reasoning and Bacharach’s team reasoning yield the same action recommendations in game theoretic settings.” (Tuomela 2013, 189). Similarly, Tuomela’s “pro-group I mode” reasoning will produce the same results as Bacharach’s team benefactor reasoning.

According to Tuomela, we-mode reasoning is not definable or functionally constructible from I-mode reasoning “because it employs a different reasoning mechanism that relies on groups as the (theoretically) basic agents of reasoning and that in some cases leads to different results than the latter.” (Tuomela 2013, 194) Similarly, groups are the theoretically basic agents in Bacharach’s team reasoning. This does not mean that they must be agents in some sense requiring group consciousness, but in the sense that the individual team reasoner *identifies* with the group in her reasoning.

She thinks and speaks of the group not as “them and me” but as “us”. If this is so then, when confronted by a decision problem, she asks herself not “what should I do to further the group interest given that the others are likely to make such and such choices?” but rather “what should we do?” But it is hard to construe “what should we do?, when it is not equivalent to the first formulation, other than as a question about the selection of a profile. This first person plural way of formulating her decision problem thus marks the difference between individualistic reasoning on behalf of the group (what benefactors do) and team reasoning. (Bacharach 1999, 135)

Tuomela sometimes treats the ‘we-mode’ as a perspectival feature of the required sort, and the switch from I-mode to we-mode as an agency transformation (rather than a mere preference transformation).

According to Tuomela, in “the case of a we-mode intention the important thing is not the specific content of the intention but *the mode of having* the intention”. (ibid., 67) This may seem to fit well in with the traditional phenomenological distinction between mode (or quality) and content (or matter) of intentional states, the idea that the same contents can be conceived in different attitudinal modes.

However, in a short parenthesis Tuomela disavows this reading. “Note that mode in the present sense is to be distinguished from the “attitudinal” mode, e.g. intending, hoping, or believing, applied to certain content.” (Tuomela 2013, 67). Tuomela repeats this clarification in a footnote, where he reminds the reader that his notion of we-modes and I-modes “should of course be kept strictly separate” from the general view that attitudes have modes and contents. (2013, 272, footnote 17)

I find this somewhat confusing. If collective intentionality is not a matter of having attitudes with collective content but a matter of the mode in which the attitude is held, then how can we understand collective intentions in a manner strictly separated from the general view that attitudes have modes and contents?

If we do not just rely on Tuomela’s informal characterisations of the we-mode but look closer at Tuomela’s quite complex *definitions* of different kinds of Tuomelian we-mode attitudes, as formulated in the 2013 book and in previous work, they seem to treat ‘we-mode’ as a generic label for several types of complex attitudes, or attitude complexes, sharing the feature that some collective notion, like ‘group’, ‘member’, ‘participation’, ‘collective acceptance’, and sometimes ‘we-mode’, somehow figure in their *content* in specific ways. The following list of necessary and sufficient conditions occurs with slight variations in several of Tuomela’s books and articles:

My analysis of we-mode we-intention can be summarily formulated as follows.

(WI) A member Ai of a collective g we-intends to do X if and only if

(i) Ai intends to do his part of X (as his part of X);

(ii) Ai has a belief to the effect that the joint action opportunities for an intentional performance of X will obtain (or at least probably will obtain), especially that a right number of the full-fledged and adequately informed members of g, as required for the performance of X, will (or at least probably will) perform their parts of X, which under normal conditions will result in an intentional joint performance of X by the participants;

(iii) Ai believes that there is (or will be) a mutual belief among the participating members of g (or at least among those participants who perform their parts of X intentionally as their parts of X there is or will be a mutual belief) to the effect that the joint action opportunities for an intentional performance of X will obtain (or at least probably will obtain);

(iv) (i) in part because of (ii) and (iii). (Tuomela 2007, 93–94, but see also e.g. 2000, 64)

Roughly, this definition explicates the we-mode in terms of the member’s intending to do his part along with beliefs about joint performance. This is in line with Tuomela’s denial of any affinities with the traditional mode/content distinction.

What I find missing in this list of necessary and sufficient conditions is the perspectival feature that plays an important role in Tuomela’s applications of the Tuomelian we-mode. As far as I can see, there is no explicit condition in this definition preventing the Tuomelian we-mode reasoner from framing the situation as a Bacharachian team benefactor rather than as a team reasoner, i.e. in terms of what I should do for us, rather than in terms of what we should do.

It may seem misleading to concentrate on the definition of a we-mode intention “in the head of an individual”. For a full-fledged collective intention and action to take place several other conditions, like criteria of collective commitment, have to be fulfilled according to Tuomela. However, what I am discussing now is not the nature of the full-fledged collective action according to Tuomela’s analysis, but the individual decision process in which Tuomela’s we-mode intention is the natural end product.^12^ Or in other terms: I want to expose the nature of the question to which a Tuomelian we-mode intention is the answer. In line with the definition above, it seems that an agent in that process must ask herself the following.

(i) Which action should I do my part of?
(ii) Which joint actions are available (will probably get a sufficient number of group members doing their parts, etc.)?
(iii) Which available joint actions are such that there will be mutual beliefs among members of the group, to the effect that the joint action opportunities for an intentional performance of those actions will obtain?

As far as I can see, rationality will not prevent a group-caring agent in the process of forming a Tuomelian we-intention from concluding that she should do her part in the joint action *B,B* when confronted with the Hi-Lo game. If I care about the group, I should do my part in *A,A* given that you choose to do your part in *A,A*, and my part in *B,B* given that you do your part in *B,B*. On the assumption that other members care about the group, *A,A* and *B,B* are both joint actions that can get support without anyone violating norms of rationality. (In a realistic scenario, *B* might even seem as a safer bet for the benefit of the group from an individual’s point of view, insofar as there is some uncertainty about whether the other members are purely motivated by concern for the group. Remember that the game as I set it up would be a clear-cut prisoner’s dilemma for self-caring agents.)

In 2013, Tuomela gives the following account of having an intention with collective content in the we-mode in an egalitarian group.

Here is a partial account of the notion of we-mode intention in an egalitarian group (i.e., one with members with equal status):

(WMI) Agent A has the intention with the collective content P in the *we-mode* in a group, g, of agents if

1. A is functioning qua member of g,
2. A’s intention presupposes that the agents in g collectively accept P as their intention (content) for satisfying the interests of g,
3. A intends to participate in the satisfaction of the intention for g, and.
4. A presupposes that the central we-mode criteria are satisfied for the participants. (Tuomela 2013, 68)

In a footnote, Tuomela adds that the “conditions of (WMI) apply to both intentions and goals, and indeed with some minor linguistic changes to all representational mental attitudes” (2013, 272, footnote 14). In the succeeding footnote to the same passage he refers back to the 2007 book for clarification of what it means to ‘act as a group member’ (condition 4) in the we-mode case. Condition (4), I take it, should be read in accordance with the definition from 2007, quoted above. In general, the 2013 book contains many references for clarification to the 2007 book and no explicit reasons are given for reading condition (4) in some new way.

Again, I see no reason why an individual in the process of forming an intention in a group context would have to frame the situation as a Bacharachian team reasoner, even if this individual is in the process of forming an intention fulfilling (1–4) above. When considering how to act in the Hi-Lo game, rationality will not prevent you from picking *B* even if the following is true.

- (WMI 1) You function as a member of our group, in trying to promote the group’s central goal, which is that we maximize the satisfaction of our initial self-caring preferences.^13^ (*B* would produce the most efficient result for us, given that I choose *B*.)
- (WMI 2) You presuppose that the agents in our group collectively will accept *B* as their intention (content) for satisfying the interests of g. That would be reasonable of them (i.e. you and me) given that the result of your deliberation is that you pick *B*.
- (WMI 3) You intend to participate in the satisfaction of the intention to perform the action for g, i.e. do *B*, and
- (WMI 4) You presuppose that the central we-mode criteria (understood in terms of the previously quoted definition from Tuomela 2007, 93–94) are satisfied for the participants.

The conditions in Tuomela’s definitions describe the required content of the group member’s intentions and beliefs. The group member intends to participate in the group action, believes that the members collectively accept the collective intention, etc. So, it seems natural to think that in this framework switching from I-mode to Tuomelian we-mode when confronted with a social dilemma should consist in a change in the *contents* of the agent’s beliefs and intentions.

The we-*perspective* is very central in Tuomela’s suggested approach to social dilemmas. My complaint is that this is not explained or captured in his analyses of the we-mode. What I find missing from Tuomela’s *definitions* (but again, not from his general treatment of the we-mode) is a clear condition of *agency transformation,* of the kind that Bacharach and Gold, and sometimes Tuomela himself, require (Gold 2013, 55).

I think that Tuomela might dismiss the possibility of relating the we-mode to the general view that intentional states have modes and contents too quickly. My suggestion is that in order to treat the switch from I-mode to we-mode as an agency transformation of the required sort, we need a *perspectival* condition on the we-mode, a condition requiring a collectivistic feature of the way in which an intentional state (in the head of an individual) is held, rather than just requiring certain kinds of contents in her goals and beliefs. The challenge, then, would be to make this collectivistic feature scientifically respectable. We should be able to give it a functional characterisation, and not just treat it as some primitive un-analysable phenomenon.

## We-Perspectives

Here is a condensed attempt to characterise a perspectival feature of the we-mode functionally.^14^

To begin with, we need to accept the general mode/content distinction. In daily speech we refer to what an intentional state is about, or directed at, as its *content*; The thing that is believed, perceived, desired, intended, etc. Let us stick to this simple informal characterisation of ‘content’. On this common sense notion of ‘content’, it is apparent that there are essential features of intentional states that do not figure in their content. That is one reason for accepting the general mode/content distinction.

Right now you see a text. A condition for that experience to count as your perception of this text (rather than as your memory, dream or wish about a text, for instance) is that this text is in front of you and that this very object is what causes your experience. But the thing perceived by you is this text, not the fact that it causes your present experience. Presumably you did not even think about the self-referentiality of your perception, let alone saw or perceived it, before I brought it up. Nonetheless, our concept of ‘perception’ is such that successful perceptions must fulfil a self-referential condition that can be described in functional terms. In other words, there are essential features of perceiving that do not belong to the content of perceptions. These belong to the attitudinal mode of perceiving. (Recanati 2009, 131–132)

A second important assumption is that we can characterise attitudinal modes functionally in terms of their success conditions. An essential element in a description of what would make a certain attitude successful is the *context of evaluation –* the context in which the content of the attitude should be evaluated. So, for your perception of this text to be veridical, the text must be present at the time and place of perceiving – i.e. here and now. The mode of perceiving is essentially such that it ties the object of the intentional state to its bearer at the time and place of bearing it. In other cases, like episodic memory, the context in which the content of the attitude should be evaluated — *cannot* coincide with the time in which the mental state is present. In such a case, the intentional content “is presented as true with respect to the situation (and the time) of the *earlier* perceptual experience.” (Recanati 2009, 141)

We categorize attitudes with similar contents under different labels (believing that p, desiring that p, etc.) because these attitudes are held in different modes – such categories are referred to as *attitudinal modes*. However, our general conceptual constraints on kinds of intentional states leave room for perspectival variations in the modes of some kinds of states. Admitting *perspectival variation* is a third essential element on the path to a functional characterisation of collective intentionality*.* Suppose I claim now that it is raining. Typically, you would regard this statement as false if it is not raining here and now. You could be wrong about that context though. Maybe I had just been phoning home and was thinking about what happens there. So, the full meaning of my utterance is richer than its content and the asserting of that content. It comes with tacit “perspectival” information about time and place for evaluation. (Recanati 2007) In a similar manner, the proper context of evaluating the content of my corresponding belief that it is raining may vary even though the intentional content of that belief is just ‘it is raining’. The point of view from which I believe determines the context of evaluation for that belief. In that sense, the belief’s content can be conceived from different perspectives.

A fourth important claim is that we must distinguish the ‘subject of intention’ of an intentional state from the ontological subject, the individual in whose head the intentional state resides. For your representation of this text to count as a perception of it, it must be the case that this text causes your experience, i.e. that it is related to *you* in a specific way. Since perceptions are self-referential they are necessarily also agent- or subject-referential. In that sense, the mode of perceiving is such that perceptions always have a subject of intention, which is a perspectival feature.

Other intentional states, like beliefs, need not have any subject of intention, since they are not essentially self-referential. The truth conditions for a belief need not refer to the believer. So, it is conceptually possible to separate the bearer of the attitude, its intentional subject (Mathiesen 2002) or ontological subject (Tuomela 2006) from the subject of intention. A subject of intention is a property of an intentional state, although some intentional states need not have any subject of intention at all. My claim here, inspired by François Recanati, is that the subject of intention is a perspectival feature of intending rather an element in its content. The reference to you that must figure in an analysis of your perception of the text does not imply that some representation of you must turn up in the content of your perception, i.e. in that which you perceive.^15^

Although I took the phrase “subject of intention” (as opposed to ontological subject) from Mathiesen and Tuomela, Recanati’s analysis of perspectival thought is what makes sense of this feature of some of our intentional states.

I believe we have reasons to accept the framework sketched above independently of questions about the we-perspective. We need a mode/content distinction and we need to distinguish between the bearer of an attitude and the intentional subject that is an essential perspectival feature of some kinds of intentional states. We must admit that the perspective from which the content of an intentional state is conceived need not be the perspective of the ontological subject at the time and place of possessing the state.

This framework also provides the conceptual space for a perspectival element in we-mode of intending, where we can have intentions held *from a collective perspective.* The suggestion is that the *subject of intention of such an intention is the collective.* Action-intentions, like perceptions, are essentially self-referential and therefore have a subject of intention. Unlike your mere desire to see the page turned, your intention to turn the page is not successful unless *you* perform the action of turning the page. This does not imply that a representation of you figures in the intentional content of the intention, i.e. in that, which you intend. The subject of intention is a perspectival feature of some types of intentional states, like perceptions and action-intentions. That feature determines the context of evaluation.

Our general conceptual constraints on success conditions admit that the context of evaluation comes apart in time or space from the actual context in which the intentional state occurs (for most intentional states except perceptions). In that sense, the spatial and temporal perspective of an intentional state — the point in time and space from which its content is conceived — need not be “here and now”.

There are no conceptual obstacles to the possibility that the context of evaluation may come apart from the actual context when it comes to agent perspectives as well. So, there is no contradiction in assuming that an intention in the head of an individual can be held from her group’s perspective.

Other conditions have to be fulfilled for collective action to take place, like some forms of communication, joint awareness or other clues prompting us to form intentions from similar perspectives, coordinate our actions etc. However, those conditions concern the genealogy and the consequences of intentions held in the collective perspective. They will be necessary in a richer account of collective action, of the kind developed by Tuomela, but they need not enter the functional characterisation of the collectivistic perspectival feature of the intention “in the head of the individual”.

Team reasoning requires the agents to conceive of their decision problem from the group’s perspective. I have suggested a way of making explicit a perspectival condition on the individual team reasoning group member’s intentional states, which is in line with that requirement.

A common taxonomy of approaches to collective intentionality groups them into three categories:

Some authors claim that collective intentionality is intentionality with a collective *content,* others seem to invoke a special *mode,* while still others claim that what’s collective about collective intentionality has to be the *subject*. (Schweikard and Schmid 2013)

The approach suggested above may appear to defy the distinction between ‘mode’ and ‘subject’ views of collective intentionality. We could say with Recanati that the agent-referential feature of some types of intentional states is an element belonging to the modes of those attitudes. My suggestion would then imply that collective action intentions and individual action intentions represent two different modes of intending – hence, a ‘mode’ approach, and perhaps a way of unpacking Searle’s allegedly primitive notion of ‘we-intentions’. However, that would be potentially misleading in virtue of the conventional use of “mode”, since it seems to place collective intending alongside with believing, perceiving and other attitudinal modes as a distinct kind of intentional state, which is not something that I want to claim.^16^

It would be less misleading to say that my approach assigns an additional, agent perspectival, feature to some kinds of attitudes, besides mode and content, and that some kinds of attitudes permit perspectival variation, not only when it comes to temporal and spatial perspectives, but also of agent perspectives. I claim that action intentions are of this kind, and leave open the question of which other types of intentional states that can be held from a group perspective.^17^ But the important thing is that there is a plausible and analysable sense in which “the subject is immanent in the attitude” (to borrow a phrase from Hans Bernhard Schmid^18^) without being part of its content.

## Some Implications

The account just sketched describes how it is possible to frame the situation from the group’s perspective, asking what we should do, in Bacharach’s sense. Switching from I-perspective to we-perspective is a genuine agency transformation: it constitutes a change of the subject of intention, albeit in the head of a single ontological subject.

An individual agent conceiving of a decision problem from the group’s perspective need not have a conception of the collective in any important sense, if “having a conception” requires that the concept in question must figure in the content of any of her intentional states. The concept of the collective is in her “frame” merely in the indirect sense that an analysis of her way of perceiving the situation must employ a (not necessarily irreducible or un-analysable) concept of collectivity. In that weak sense, the team reasoner must have or possess a collectivistic attitude. So, the we-perspective condition does not demand very sophisticated conceptual resources from the agent.

By denying that collectivity is an element of the content of collective intentions the account does not exclude the possibility that all or some collective actions have characteristic phenomenal features – what some refer to as “a sense of we-ness” (Pacherie 2012, 343). As Recanati says, “there is absolutely no reason to consider that phenomenology supervenes on *explicit* content. The mode also contributes to the phenomenology”. A scene represented in a memory may e.g. be “felt as past” (Recanati 2009, 141–142). In a similar manner, it is possible – but not essential to the proposed analysis - that an intention held from the collective perspective can be felt as ours.

According to Bacharach, the change between individualistic reasoning and team reasoning is a psychological matter, determined prior to rational choice. By contrast, Tuomela takes the “common-sense view that not only can the mode be intentionally selected by an agent but in some cases it can also be rationally intentionally selected”. (Tuomela 2013, 195) But from whose perspective would it be rational to choose the collective perspective? As Leo Townsend notes in a recent review, this seems to imply a meta-frame encompassing both I-mode and we-mode, which seems highly problematic. (Townsend 2015, 183–187)

I suspect that Tuomela’s optimism about the agent’s rational capacity in this respect has something to do with the mentioned tension between his applications of the we-mode and his explicit analyses. It does not seem too implausible to think that if collectivity was something that merely figured in the *content* of our attitudes, then we would at least to some extent be able to switch deliberately between I-thinking and we-thinking. We might think of a set of people to which we belong as a mere collection of individuals, or as a genuine collective in a holistic sense. Apparently we have that choice when we look at other collections of things, like beehives: we can treat them as single units of activity or as several distinct individuals with special relations between them, depending on what is most useful for our purposes.

However, we do not seem to have the same capacity for voluntary shifts of attitudinal modes or perspectives. I do not deliberately switch from fearing that p to believing or hoping that p.^19^ Admittedly, there are various well-known indirect ways in which we can manipulate ourselves into coming to believe something or stop fearing something, by seeking certain kinds of evidence, avoiding certain lines of thought, etc. But generally, shifts in attitudinal modes are prompted by situations and inputs, even if we are capable of manipulating these inputs to some extent. The same appears to hold for perspectival differences within an attitudinal category. It seems that I cannot directly choose how distant in time the object of a certain episodic memory should appear to me, for instance.

These are psychological generalizations that may be contested. More essential in the present context is that permitting rational meta-considerations about *agency* perspectives seems problematic for formal reasons. Pacherie elaborates the point:

I suspect that at bottom what Bacharach really opposed was the idea that the adoption of a frame can be a matter of *rational* choice. His reluctance appears founded, if we consider what a meta-level version of the Prisoner’s Dilemma looks like. /…/ The payoff matrix of the meta-level version of the Prisoner’s Dilemma is exactly the same of the payoff matrix of the original Prisoner’s Dilemma. We are thus back to square one. If the players ask the question “What question should I ask?”, the answer is that the I-question should be chosen, but if they ask the question “What question should we ask?”, the answer is the We-question.

Thus, on pain of infinite regress, the cost to be paid for preserving the rationality assumptions of the classical game theory while resolving its puzzles, is in accepting that the adoption of one mode of reasoning over another cannot be a matter of rational choice. (Pacherie 2011, 186–187)

Understanding collective intentionality partly in terms of a perspectival feature of intentional states is in line with Bacharach’s insistence that it is a brute fact that some situations prompt the shift of frames that constitutes an agency transformation, rather than a matter of rational choice. It seems plausible that we may promote reasoning from the collective perspective indirectly, by affecting the conditions for group identification etc., but in general I have to be more sceptical than Tuomela about the prospects of rationally choosing to view a choice situation from the group’s viewpoint.

## Summary

In experimental settings, people’s proneness to cooperate in social dilemmas has often been tested with cash as payoffs. People then tend to cooperate to a varying extent. (See e.g. Colman et al. 2008) If the game is a prisoner’s dilemma, and they each want as much money for themselves as possible, cooperation implies that they are irrational in the simple sense that they fail to maximize whatever it is that they want to maximize. However, it is possible that people sometimes cooperate because they are team benefactors who want as much money as possible for the group within which they have to function. In that case, they no longer conceive of the choice situation as a prisoner’s dilemma – the relative size of payoffs as determined by their preferences do not correspond to the amount of cash they will each receive. So, their behaviour is no anomaly within game theory.

As we have seen from discussion of the Hi-Lo game, being a team benefactor will not always guarantee the collectively attractive outcome. The reason for this is that a team benefactor is still framing the situation from an individual perspective, and even if she wants the best for her team, that is not sufficient to make her act “on the best feasible combinations of actions for all the members of her team” (Bacharach 2006, 121). The team benefactor is doing her best for the team, given how others will act, and that may produce a collectively suboptimal result.

By contrast the team reasoner works out the best feasible combinations of actions for all the members of her team and then infers her part in it. While being a team benefactor is a matter of having certain team-directed preferences, the concept of team reasoning need not involve any substantial assumptions about what exactly the team reasoner wants or prefers. The important thing is that given the preferences in play in the specific choice situation, the individual team reasoner asks “What should we do?” rather than “What should I do for us?” As Bacharach says, this requires a certain kind of *agency transformation* that might seem puzzling from an action theoretical point of view.

I have examined and rejected two possible ways in which this agency transformation could be understood; In terms of Bratman’s theory of shared intention, where the group is supposed to figure in the content of the individual member’s motivation, and in terms of Tuomela’s “we-mode” of intending, where I found the perspectival element of agency transformation missing from the explicit analyses. My positive contribution is a characterization of this form of agency transformation “in the head of an individual” in functional terms, inspired by Recanati’s theory of perspectival thought. I claim that it is possible to frame the situation from the group’s perspective, and that switching from I-perspective to we-perspective is a genuine agency transformation: it constitutes a change of the subject of intention, albeit in the head of a single ontological subject.

It should be noted that this is a claim about one essential but particularly elusive condition for team reasoning. The agency transformation described allows team reasoners to frame situations that would have been genuine social dilemmas for team benefactors or ordinary individual reasoners in a different way. For team reasoning to result in decisions and actions, other conditions may have to be fulfilled as well – causal, epistemic etc. The question of how to spell them out will have to be left open here.

## Footnotes

1 It is not obvious that this will suffice to ensure cooperation, though. See Jiborn and Rabinowicz 2003.

2 For a clarifying discussion of the relation between realism/formalism and explanatory or normative force of decision theory, see Hansson 1988, 145–146)

3 Bratman’s and Tuomela’s accounts share a commitment to what might be called ”mental individualism”, i.e. the view that intentional states, including collective ones, reside ”in the heads of individuals” to use a phrase from Searle. As pointed out by an anonymous reviewer, cooperation in potential prisoner’s dilemma situations might also be explained within a theory admitting genuine group intentionality – Pettit’s ”Groups with Minds of their Own” (2003) might be a case in point. However, that would not capture the agency transformation and the vacillation between frames that explains how a specific social choice situation with given payoffs can be conceived of in two distinct ways by a single player (who might even be a group agent). Moreover, what I am interested in here is the conditions that would have to be fulfilled for team reasoning to develop, rather than what happens once a full-fledged collective intention is in place. For such reasons I will not discuss those sorts of accounts here. See also footnote 14.

4 Elisabeth Pacherie thinks that the substantive theory of collective intentionality that “Bacharach is closest to in spirit is probably Searle’s” (2011, 187) but she shares the worry of many philosophers about Searle’s brief and incomplete characterization of ‘we-intentions”. (ibid. 178) My proposal about a collective perspective *might* be read as a way of unpacking Searlean we-intentions in relation to team reasoning (see section 5).

5 In the illustration that gave name to the dilemma in the fifties, the numbers represent years of prison sentences, thereby illustrating a reversed order of preferences. (Steven Kuhn 2014) In my matrix higher numbers represent more desirable outcomes. Think of the matrix as representing the preferences of two lazy persons placing themselves in a rowing boat with two pairs of oars, both desiring to get across a lake. As John Mackie points out, this classical example from David Hume’s *A Treatise of Human Nature* can be interpreted as a Prisoners’ Dilemma, where the best outcome for each (4) is achieved when the other does most of the rowing even if that means crossing takes somewhat longer, the second best for each (3) will result from both making a reasonable effort, the second worst for each (2) is when both make a minimal effort, and the worst for each (1) would be if he ended up doing all the hard work. (Hume 1739, 3:2:2, Mackie 1980, 88)

6 ”Rationality” is used in the game-theoretical sense*,* and the central notion is *Nash Equilibrium.* A set of strategies for all the players in a game is a Nash Equilibrium if it is such that no individual player can raise her payoff by unilaterally changing strategy, given the strategies of the other players. In the Prisoner’s Dilemma there is only one Nash Equilibrium while there are two in the Hi-Lo game.

7 Team benefactors do not have to be preference-utilitarians with respect to their group, of course, and the game would be transformed quite differently for a team benefactor with, say, strongly egalitarian preferences for her group.

8 Tuomela says that ”[w]hat kind of account we get or could get for cooperative act types from the Bratmanian account seems to be an open problem. Would he after all accept that J in the above account could be instantiated also by a cooperative act type—creating circularity”? (2013, 159) I think that Bratman would accept that J sometimes could be instantiated by an essentially cooperative action type. What is needed to avoid circularity is merely that such references are eliminated from the conceptual analysis of ‘cooperative action’. This should not be more problematic than that some individual action types are essentially intentional, which does not force us to refer to intentions in the analysis of ‘intentional action’. Arguably, *voting* is an action-type of this kind – see Steward 2012, 94 – and *lying*, as opposed to merely saying something false, might be another.

9 Similar conclusions about the individualistic limitations of Bratman’s theory in relation to team reasoning have been drawn by Pacherie (2011, 187–188), Bardsley (2007, 150), and Gold and Sugden (2007, 116)

10 Van Hees and Roy argue that future-directed intentions understood in terms of Bratman’s planning theory may explain focal points in decisions and games, and that given reasonable rationality constraints on intentions (e.g. that intentions will focus feasible outcomes and that feasibility considerations include what would be rational in terms of utility for other players), two “intention-rational” players will intend to realize the Pareto-optimal outcome in a Hi-Lo game. (2007, p. 214–15) I will not attempt to assess this strategy here but it should be noted that the approach of van Hees and Roy merely relies on Bratman’s characterisation of individual planning agency and contains no claims about his theory of shared intention in relation to social dilemmas. So, it differs radically from the strategy considered by Tuomela in relation to Bratman’s theory. Moreover, as van Hees and Roy admit, individual intentions fulfilling the proper rationality constraints are still such that an agent “will always intend to realise one or more of *his* best feasible outcomes” and that intentions “are forced into an *individualistic* maximising behaviour” (p. 217), which leads to counter-intuitive results in other game theoretic dilemmas. They mention the possibility of weakening the relevant axioms ”so as to give a less individualistic interpretation” (p. 217) but do not pursue that further.

11 Bratman could respond to this in two ways. First, he could remind us that he never claimed that there is a necessary continuity between individual and social rationality, just that *normally* there is such continuity. Second, he could bite the bullet and question the presumption that social rationality norms dictate something to players confronting a Hi-Lo game. Presumably, norms of social rationality should govern social reasoning in accordance with the possible aims of co-operating agents.

12 Similar points have been made with respect to Margaret Gilbert’s theory of ”joint commitment” in relation to team reasoning, as well as about Bratman’s theory (Pacherie 2011, 188, Bardsley 2007, 154). Tuomela’s, Bratman’s, and Gilbert’s analyses of collective action provide descriptions of how a group functions *once the group has formed a collective intention,* i.e. a Gilbertian joint commitment, a Bratmanian shared intention, or a Tuomelian we-mode intention. But they do not provide an analysis of the required form of motivation that would allow this formation of a collective intention to get started when individual agents face a genuine social dilemma. Pacherie discerns a related problem in Sugden’s assurance view of team reasoning. (Pacherie 2011, 188–189)

13 “Acting as a group member in the *core* sense is to act intentionally within the group’s realm of concern, promoting (furthering) the satisfaction and maintenance of the ethos—the central, constitutive goals, values, standards, beliefs, norms, and/or elements of the history of the group. The ethos is supposed to give the members a reason for action when acting as group members.” (Tuomela 2007, 23)

14 Elements of this approach were briefly sketched in Petersson 2015.

15 By contrast, John Searle assumes that the necessary reference to the agent must appear in the *content* of the perception, and this follows from his wide definition of ‘content’ according to which “the *specification* of the content is already a *specification* of the conditions of success”. (Searle 1983, 11–12) According to François Recanati, Searle makes himself guilty of what Recanati calls “the fallacy of misplaced information” when he places a self-reference in the content of e.g. perceptual states. “The self-referential condition is not determined by the propositional content of the state, but by its mode. It follows that the propositional content of a perceptual state is not self-referential, even if its overall truth-conditions are.” (Recanati 2007, 129).

16 I am grateful to one of this journal’s reviewers for helpful suggestions concerning the categorization of my view.

17 Another candidate for application of this approach may be *joint attention,* which does not seem to require very sophisticated conceptual abilities. Nevertheless it seems common to analyse the phenomenon in terms associated with *mutual* attention. Subjects “actually attend to each other as subjects capable of attending” (Brinck 2004, 13). A perspectival understanding would be less demanding.

18 Hans Bernhard Schmid “The Duty to Know What We are Doing Together”, talk at ENSO IV September 2015.

19 Classical arguments against the view that we have direct voluntary control of our beliefs were put forward by Bernard Williams (1970). A few philosophers defend direct doxastic voluntarism but the position I presuppose here appears by far to be the most widely held – that we can affect our beliefs indirectly but not directly. http://www.iep.utm.edu/doxa-vol/
